# Precision Medicine for Diabetic Retinopathy: Integrating Genetics, Biomarkers, Lifestyle, and AI

**DOI:** 10.3390/genes16091096

**Published:** 2025-09-16

**Authors:** Connor Kaurich, Neha Mahajan, Ashay D. Bhatwadekar

**Affiliations:** 1 Indiana University School of Medicine, Indianapolis, IN 46202, USA; cskauric@iu.edu; 2 Department of Ophthalmology, Eugene and Marilyn Glick Eye Institute, Indiana University School of Medicine, Indianapolis, IN 46202, USA; nehmaha@iu.edu

**Keywords:** diabetic retinopathy, personalized medicine, precision medicine

## Abstract

Diabetic retinopathy (DR) is a common sight-threatening complication of diabetes. Overall, 26% of the 37 million Americans with diabetes suffer from DR, and 5% of people with diabetes suffer from vision-threatening DR. DR is a heterogeneous disease; thus, it is essential to acknowledge this diversity as we advance toward precision medicine. The current classification for DR primarily focuses on the microvascular component of disease progression, which does not fully capture the heterogeneity of disease etiology in different patients. Further, researchers in the field have suggested renewed interest in improving diagnosis and treatment modalities for personalized care in DR management. Moreover, genetic factors, lifestyle, and environmental variation strongly affect the disease outcome. It is important to emphasize that various ocular and peripheral biomarkers, along with imaging techniques, significantly influence the diagnosis of DR. Therefore, in this review, we explore the heterogeneity of DR, genetic variations or polymorphism, lifestyle and environmental factors, and how these factors may affect the development of precision medicine for DR. First, we provide a rationale for the necessity of a multifaceted understanding of disease etiology. Next, we discuss different aspects of DR diagnosis, emphasizing the need for further stratification of patient populations to facilitate personalized treatment. We then discuss different genetics, race, sex, lifestyle, and environmental factors that could help personalize treatments for DR. Lastly, we summarize the available literature to elaborate how artificial intelligence can enhance diagnostics and disease classification and create personalized treatments, ultimately improving disease outcomes in DR patients who do not respond to first-line care.

## 1. Introduction

Diabetic retinopathy (DR) is a microvascular complication associated with diabetes and is a leading cause of disability in working-age adults [1]. DR incidence in the United States remains high, with 5% of the 37 million Americans with diabetes having vision-threatening diabetic retinopathy (VTDR) [2]. Globally, rates of VTDR are expected to increase from 29 million people in 2020 to 45 million in 2045, representing an increasing public health threat and burden on patients worldwide [3]. For individuals without access to care or an adequate treatment regimen, progression to more severe forms of DR and, eventually, central vision loss is a hallmark of DR’s natural history [4]. In current practice, DR progression is classified according to the international clinical diabetic retinopathy (ICDR) severity scale [5]. The ICDR scale categorizes DR into five stages based on retinal findings observed during fundoscopic examination, while the presence or absence of diabetic macular edema (DME) is evaluated independently [6]. While microvascular changes predominantly define DR etiology and progression, recent studies have suggested an update of our current understanding of DR pathophysiology [7]. Accordingly, others in the field have advocated for an updated staging system for diabetic retinal disease, showing that the ubiquitous ICDR scale does not adequately evaluate the neural retina, key molecular and inflammatory pathways, or the peripheral retina [8]. An enhanced understanding of DR pathophysiology through the analysis of biomarkers and imaging modalities can help improve DR diagnosis, disease classification, and personalized treatment strategies, ultimately leading to improved patient outcomes.

Precision medicine has gained mainstream attention since 2015, when President Barack Obama announced the Precision Medicine Initiative with the goal of “bringing us closer to curing diseases like cancer and diabetes” [9]. Precision medicine aims to provide better medical and health recommendations for an individual using information like genetics, environmental factors, and lifestyle, with the goal of improving prediction, prevention, diagnosis, and/or treatment of the disease. The core tenet of precision medicine involves taking individual patient variability into account for the prevention and treatment of disease [9]. Likewise, since 2018, the Food and Drug Administration (FDA) has defined precision medicine as an approach that considers the differences in patients’ genes, lifestyles, and environments for the prevention and treatment of disease [10]. While exact definitions vary slightly from source to source, the two main goals of precision medicine remain the same: subtyping diseases and tailoring treatments to that specific disease subtype [11]. DR is the most common microvascular complication of diabetes. Considering the diversity of DR, a precision medicine approach could help patients by combining the intricate pathophysiology of DR with current classification systems, while also including personal genetic, lifestyle, and environmental factors to improve prevention, diagnosis, and personalized treatment strategies. This review covers the following related to precision medicine for DR: heterogeneity of DR, genetic, lifestyle, and environmental factors related to DR, biomarkers for diagnosis and prognosis, and applications of AI for data integration.

## 2. Heterogeneity of DR

While DR is mainly defined as a microvascular disease [12], there has been a significant motivation to uncover more about the pathophysiology of DR. As previously mentioned, many studies have suggested an update to the current staging methods used for diagnosing and monitoring DR [7,8,13]. To contextualize the need for an updated staging system, we will first review the three major phenotypes contributing to the heterogeneity of diabetic retinopathy. We will then examine how assigned sex at birth (ASAB) and race further shape disease risk, progression, and patient outcomes.

### 2.1. Neurodegenerative Phenotype of DR

Neurodegeneration in the retina, defined by a loss of ganglion cells and glial dysfunction (inner neural retinal degeneration), has been emphasized in DR for at least the past 60 years [14]. However, the exact interplay between microvascular changes and neuronal function is not well defined either temporally or spatially. Researchers have begun to define the retina as a neurovascular unit, composed of neurons, glial cells, and blood vessels [15], to better understand the normal and pathological function of the retina. Recent studies have attempted to uncover the relationship between the onset of microvascular symptoms (as measured by the ICDR scale) and neuron layer changes in the retina. While there is ambiguity in the exact temporal relationship between neurodegeneration and microvascular changes, most clinical studies report that functional retinal changes (as measured by electroretinogram (ERG)) precede vascular abnormalities in patients [16,17]. Individuals without clinical signs of DR represent a therapeutic opportunity for treatment because this group represents most patients and because they respond better to intensive therapy [14]. Although most individuals with type 2 diabetes (T2D) exhibit signs of neurodegenerative changes prior to microvascular alterations (somewhat, “clinically significant” DR), around 30% of individuals with T2D with microvascular changes diagnosed through fundoscopic image do not show any signs of neurodegeneration, even as their DR progresses microvascularly [18]. In some studies, authors found that individuals with clinical diabetes, but without clinically significant DR, showed progressive loss of the nerve fiber layer (NFL) as measured by OCT [16,17,18,19]. Predominant or independent phenotypes of either neuroretina degeneration or microvascular complications in DR could serve as a basis for subtyping individuals, thereby helping to unravel the heterogeneity of the disease. Furthermore, individuals with predominantly neuroretinal changes may benefit from an increased frequency of examinations and, potentially, earlier intervention. More research is needed to clarify the disease course for neuroretina-dominant DR, as this may inform treatment practices and prevention strategies in the future. Additionally, further research is needed to characterize disease progression for individuals with microvascular-dominant DR, as they may have a distinct prognosis and response to treatment. Phenotypic independence could be integrated into a precision medicine approach to ultimately treat the right patient, at the right time, with the right therapy.

Diabetes type may also need to be considered for the use of functional ERG data as a predictor of the onset of DR; while neuroretinal degeneration is commonly observed in pre-DR and early DR in both type 1 diabetes (T1D) and T2D, individuals with T1D may be at an increased risk for early neuroretinal degeneration preceding microvascular DR, as evidenced by functional ERG studies in adolescent individuals with T1D [20,21]. Studies have shown that in type 1 diabetes, neuroretinal alterations of a smaller magnitude on ERG are predictive of a risk of clinical DR equivalent to that conferred by larger ERG abnormalities in type 2 diabetes [17]. In a cross-sectional study by Reis et al., T1D subjects were separated into two groups based on the presence or absence of any blood–retinal-barrier (BRB) permeability (to detect preclinical DR vascular changes and mild nonproliferative (NPDR)) measured by vitreous fluorometry [22]. Authors found that both groups exhibited reduced mfERG amplitudes as compared to age-matched controls, indicating functional neuronal deficits independent of BRB permeability [22]. Additionally, neural deficits seem to correlate with BRB permeability in the leakage group, indicating that neurodegeneration superimposed onto microvascular changes could lead to worse outcomes clinically [22].

### 2.2. Microvascular Phenotype of DR: Historical Perspective and Natural History of Disease

**Figure 1:**
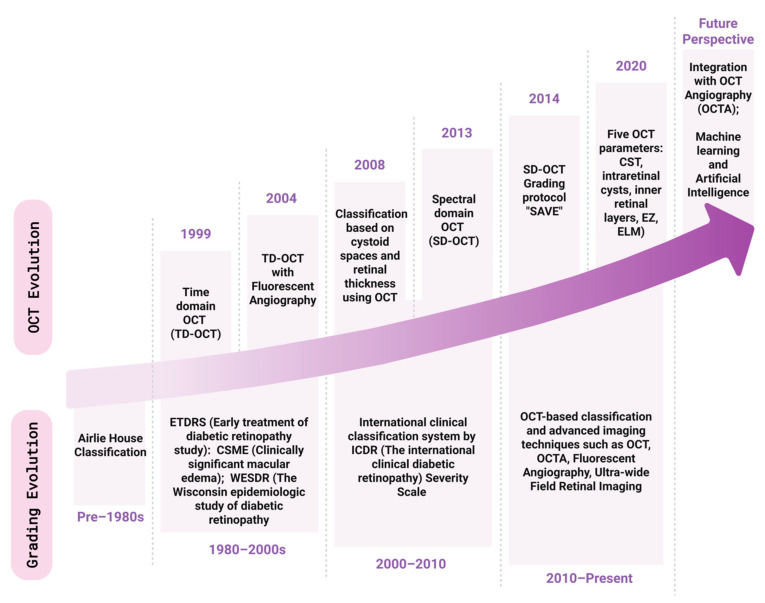
Progression of OCT and grading classification system for DR and DME. Over the years, both scientific and clinical efforts have been undertaken to improve the diagnosis of DR and DME. OCT imaging, one of the earliest techniques, has progressed swiftly and is one of the routinely used imaging methods in clinics for DR and DME categorization.

The microvascular phenotype represents the canonical and most well-studied presentation of DR. As described in the introduction, DR has long been known as a microvascular disorder driven by hyperglycemic conditions. Since the introduction of the direct ophthalmoscope, descriptions of retinal hemorrhage and vascular proliferation in diabetic patients have been widespread [23]. By 1890, Julius Hirschberg created the first classification system for DR, subdividing disease progression into three parts, describing a punctate form in the posterior pole, a hemorrhagic subtype, and retinal infarction, effectively capturing both hemorrhagic and proliferative characteristics of advanced DR [5]. Eventually, in 1966, Lee et al. created a DR classification system that is more similar to the modern ICDR scale, utilizing grading of individual lesions on a scale of 1 to 5, with each lesion classified as one of four major lesion types (angiopathy, exudates, proliferative retinopathy, and vitreous hemorrhage) [24]. While this method of lesion severity grading was time-consuming, based on hand-drawn fundoscopic images, it represents the first categorical grading of individual lesion severity, paving the way for future grading systems with automated fundoscopic imaging. Later, the Early Treatment of Diabetic Retinopathy study (ETDRS) scale was developed, clarifying our descriptions of microvascular changes from Lee et al. to be more detailed. The ETDRS integrated individual lesion characteristics (hemorrhages/microaneurysms, venous bleeding and loops, hard exudates, intraretinal microvascular abnormalities, and neovascularization) and graded them on a scale of 1 to 5. Then, individual lesion scores were used for a global assessment of the eye, staging overall disease severity on a 14-level scale [25]. Additionally, ETDRS defined clinically significant macular edema (CSME), separately, based on the thickening of retinal tissue or exudate formation and relative distance to the center of the macula [5]. Therefore, the ETDRS scale was able to build on past categorizations, from Hirschberg to Lee, to develop a detailed, prognostic tool for DR progression. While the ETDRS was rigorously validated as an effective predictive tool [26], there was limited widespread adoption because of the time-intensive nature of using this staging system. For this reason, there was motivation to create a grading system that could be widely adopted internationally by ophthalmologists and primary care doctors alike. To make grading more efficient, clinicians from 14 countries met in the early 2000s to discuss a more practical version of the ETDRS scale for daily clinical use [5]. At this meeting, clinicians defined the ICDR scale, which effectively distilled the 14 severity ratings from the ETDRS into a 5-point severity scale: this scale includes (0) no apparent retinopathy (1) mild nonproliferative retinopathy (microaneurysms only) (2) moderate nonproliferative retinopathy (microaneurysms, dot or blot hemorrhage, cotton wool spots) (3) severe nonproliferative retinopathy (>20 hemorrhages in all four quadrants, venous bleeding in two or more quadrants, intraretinal microvascular abnormality, no sign of proliferative retinopathy) (4) proliferative retinopathy (neovascularization or vitreous hemorrhage) [6]. Similar to the ETDRS scale, the ICDR scale also evaluates the presence or absence of macular edema on a separate axis but includes severity scales for DME based on fundoscopic imaging (mild, moderate, and severe DME based on the location of thickening with respect to the fovea) [6]. Figure 1 highlights major developments in microvascular grading (bottom) as well as advancements in OCT imaging (top) for the grading of DME. A discussion of OCT imaging for DME grading can be found in Section 2.3.

As discussed, there is a long history of defining DR in terms of the vascular abnormalities that are observed. While mechanistic studies involving DR continue to uncover varied disease pathophysiology and presentation, microvascular classifications are widely used due to their effectiveness, widespread adoption, and ease of use. These categorizations have a high level of interobserver agreement as well as a high sensitivity and specificity, enabling clinicians to accurately diagnose, measure, and track disease progression. There have been some proposed developments enabling a multidimensional diabetic retinal disease severity scale that addresses retinal, neural, and vascular pathology as well as systemic factors such as the type of diabetes, glycemic control, blood pressure, presence of renal disease, and anemia [8]. Such a scaling system could provide the greatest benefits to individuals with diabetes, offering greater precision in diagnostic care for individuals with DR.

### 2.3. Edematous Phenotype of DR

DME is a major complication of DR that can occur at any stage of disease progression. It is characterized by the accumulation of extracellular fluid within the macula due to the breakdown of the blood–retinal barrier. This fluid may collect in the form of intraretinal cysts, diffuse thickening, or subretinal fluid [1,27]. As discussed in prior sections, the presence of DME is evaluated in the ETDRS scale and the current ICDR scale. The ETDRS scale defines clinically significant macular edema (CSME) as “(1) Thickening of the retina at or within 500 microns of the center of the macula; or (2) hard exudate at or within 500 microns of the center of the macula associated with thickening of the adjacent retina; or (3) a zone of retinal thickening one disc area or larger, any part of which is within one disc diameter of the center of the macula” [25]. On the other hand, the ICDR scale defines the absence or presence of retinal thickening or lipid deposition in the macula, with the addition of three severity levels if DME is present: mild (retinal thickening or lipid deposits in posterior pole but distant from macula center), moderate (retinal thickening or lipid deposits approaching the center but not involving the center of macula), and severe (thickening or lipid deposits involving the center of macula) [6]. This two-tiered distinction, first involving a binary decision and second involving scaled grading of DME is meant to improve access for clinicians who may only be able to perform direct ophthalmoscopy.

While the presence of DME is evaluated in both major classification systems (ETDRS and ICDR), innovations in ophthalmic diagnostic techniques, such as OCT (optical coherence tomography), OCT angiography (OCT-A) (summarized in Figure 1), and ERG, have elucidated varying DME phenotypes that are not typically seen with standard imaging [13]. With more advanced examination (either through stereoscopic fundus imaging or slit lamp examinations), specified grading can be determined [6].

OCT may offer a more quantitative and systematic way of detecting DME, without relying on subjective clinical interpretation as in the ETDRS and ICDR. The increased use of OCT for detecting DME has led to two major classification systems for phenotypic characterization. Researchers in Japan characterized DME morphology based on three distinct changes seen in time domain OCT (TD-OCT) imaging: sponge-like diffuse retinal swelling, cystoid macular edema, and serous retinal detachment [28]. Several other studies have used this same method of DME subtyping based on OCT characteristics to describe distinct DME morphology [29,30,31]. Additionally, a recent study from 2020 by the European School of Advanced Studies in Ophthalmology (ESASO) describes an updated DME classification system that incorporates seven variables (TCED-HFV) determined by OCT [32]. The four most important variables are as follows: thickness (central subfield thickness), cysts (size and number of intraretinal cystic spaces), ellipsoid zone (integrity of inner/outer segment photoreceptor junctions), and DRIL (presence of disorganization of inner retinal layers). These variables can be combined to determine four clinical stages, grading severity from early DME to atrophic DME.

### 2.4. Contribution of Assigned Sex at Birth on DR Heterogeneity

ASAB is a known risk factor for diabetes and its complications [33]. However, there is debate in the medical literature on the influence of ASAB on DR. In one meta-analysis from 2012, researchers analyzed data from nearly 23,000 individuals from 35 studies and found that there was no discernible difference in the prevalence of DR, proliferative (PDR), DME, or VTDR based on sex [34]. Similarly, a systematic review published in 2019 by Sabanayagam et al. found that, in seven out of the eight studies analyzed in the review, there was no significant difference in the incidence of DR based on ASAB [4]. It is important to note that there are some limitations to the studies included in this review. For example, the study by Sabanayagam et al. characterized the incidence of DR, and as the authors note, there was limited data from “developed” nations that have better mechanisms for early screening and detection, potentially decreasing the generalizability of study results [4]. While the criteria in these two studies differ significantly, they add additional evidence that ASAB may not stratify DR risk of onset and progression. Further, a systematic review and meta-analysis by Lundeen et al. recently found that the prevalence of DR and VTDR is higher in men (28.1% of diabetic men with DR versus 24.43% of diabetic women; 5.34% of men with VTDR versus 4.72% of women) [2]. In this study, results for sex were adjusted for age and race. However, neither of the results was deemed significant, as the 95% uncertainty intervals (UI) overlapped for men and women for both DR and VTDR prevalence. A meta-analysis analyzing the prevalence of DR in pediatric T2D, performed by Cionna et al., showed that DR prevalence was higher in men than women, but this difference was not significant (*p*-value = 0.51) [35]. From the available high-quality evidence reported in the literature, there is still uncertainty involving ASAB as a risk factor for DR onset and progression. Overall, the literature seems to suggest that there is no relationship between assigned sex at birth and DR risk, and, therefore, it is uncertain if ASAB should be considered a contributing factor for DR heterogeneity.

### 2.5. Influence of Race on DR Heterogeneity

While the U.S. census defines race and ethnicity separately, with race defined as White, Asian, Black, American Indian, or Native Hawaiian, and ethnicity as Hispanic/Latino or non-Hispanic/Latino, these identities can overlap (e.g., Black and Hispanic, White and Hispanic) [36]. Ideally, clinical research studies would first analyze outcomes by race and then stratify by Hispanic heritage. However, the studies discussed below treated Hispanic ethnicity as a mutually exclusive category, grouping all individuals of Hispanic origin together regardless of their racial identity [36], resulting in inconsistencies when assessing the effect of race and ethnicity on DR.

Just as ASAB influences the risk of diabetes and its complications, racial and ethnic background also play an important role in determining the risk of diabetes. A 2019 study published in JAMA showed that non-Hispanic Black, Hispanic, and non-Hispanic Asian groups had a higher prevalence of undiagnosed and diagnosed diabetes compared to White participants [37]. An analysis of a large representative sample of diabetic participants based on National Health and Nutrition Exam Surveys (NHANES) data from 2005 to 2008 shows that the prevalence of DR is higher in non-Hispanic Black populations compared to non-Hispanic White individuals (38.8% of Black patients with diabetes were found to have DR versus 26.4% of non-Hispanic White individuals, *p* = 0.008) [38]. Additionally, Hispanic participants were found to have a DR prevalence of 34.0% (*p* = 0.008 when compared to White participants) [38]. Furthermore, the prevalence of VTDR was significantly higher in non-Hispanic Black and Hispanic participants when compared to White participants (*p* = 0.006) [38]. Authors of this study also performed multivariate logistic regression analysis on this data set to analyze race, along with many other variables such as HbA1c, diabetes duration, and age as independent risk factors for DR and VTDR reported as an odds ratio (risk relative to White participants). It was reported that race alone was not a reliable risk factor for the development of DR, but it was for the development of VTDR for Black and Hispanic participants [38]. Similarly, in a prospective cohort study involving over 6000 participants, it was found that the four-year incidence and progression of DR and DME in Hispanic patients was greater compared to White patients after accounting for population demographics in which the sample was taken [39]. Additionally, a meta-analysis including 59 population-based studies from around the world, using multivariate regression (adjusting for study-level characteristics like habitation type, response rate, and diagnostic method), found that individuals with diabetes of Middle Eastern and North African descent (MENA) and of Hispanic descent had a higher prevalence of DR relative to participants of Asian descent [3]. On the other hand, a meta-analysis by Lundeen et al. reported that the prevalence of DR for Hispanic individuals and Black individuals was insignificantly greater than that of White participants [2]. However, estimates for VTDR showed that Black participants (8.66% uncertainty interval: 5.91–12.97) had a significantly higher prevalence of VTDR compared to White participants (3.55% uncertainty interval: 2.22–5.41) [2].

Overall, the story of race as a risk factor for DR is nuanced, and the literature suggests that race can only be used as a significant, independent risk factor for vision-threatening DR, as evidenced by covariate analysis controlling for systemic factors like A1c, hypertension, and duration of diabetes.

## 3. Genetics

Monogenic diseases like cystic fibrosis have been greatly impacted by the development of the Human Genome Project, where genetic information has helped with the progression of precision medicine. On the contrary, complex diseases like diabetes and obesity not only depend on the genomic and molecular markers but also on lifestyle and environmental factors. Genetic factors influence the onset, pace of progression, and severity of DR; therefore, it is crucial to study the genetic factors before diving into the reality of precision medicine for DR. An individual’s genetics strongly affect the associated biochemical pathways listed as the contributing factors for DR development. A few factors of importance are vascular endothelial growth factor (VEGF), advanced glycation end products (AGEs), sorbitol accumulation, oxidative stress, and inflammation. Importantly, DR is a polygenic disease where genetic factors contribute to 25–50% of the risk of disease development. Genetic polymorphism studies based on single-nucleotide polymorphism (SNPs) have uncovered the regulation of these important pathophysiological metabolic pathways by SNPs.

### 3.1. Genes and DR

Several studies have unveiled various genes and their polymorphic variants (SNPs) located on different chromosomes potentially participating in the progression of DR (Table 1). Chromosome 1 has the greatest number of genes (*SELP*, Methylenetetrahydrofolate reductase (*MTHFR*), nuclear valosin-containing protein-like 2 (*NVL*), C-reactive protein (CRP) followed by chromosome 7 (interleukin-6 (IL-6), endothelial nitric oxide (*eNOS*), aldolase reductase (*AR*), plasminogen activator inhibitor-1 (*PAI-1*))). On the other hand, chromosome 6 has the vascular endothelial growth factor (*VEGF*) gene, chromosome 17 has the angiotensin-converting enzyme (*ACE*), and chromosome 19 has the apolipoprotein E (*APOE*) and intercellular adhesion molecule-1 (*ICAM-1*), which have been studied the most for DR progression and are very well summarized by Sienkiewicz-Szłapka et al. [40]. As mentioned earlier, these genes can significantly alter metabolic pathways, thereby changing the onset, pace, and severity of DR progression.

#### 3.1.1. *SELP*

*SELP*, a gene encoding P-selectin, is present on chromosome 1. Various SNPs associated with *SELP* have been reported not only for DR but for a wide range of inflammatory diseases, including atherosclerosis, ischemic stroke, CAD, and T2D. P-selectin is a cell adhesion molecule present in endothelial cells. It is believed that P-selectin plays a crucial role in leukocyte adhesion, leukostasis, and microangiopathy [123]. Importantly, leukocytes provoked by P-selectins also facilitate the proliferative damage of the retinal vasculature; thus, it is suspected that P-selectin has the potential to participate in both NPDR and PDR development. Numerous clinical studies around the world have shown promising results. In an international study on T2D individuals, three SNPs of the *SELP* gene (rs6128, rs6133, and rs3917779) were found to be associated with DR [108]. However, this association was limited only to the European American population and was not observed in any other ethnic group. Another study conducted on an ethnically homogeneous group of African American T2D individuals reported an association between the genetic variant of the *SELP* gene rs6128, plasma P-selectin levels, and DR outcomes [109]. Another study from Iran on PDR patients found no polymorphism for rs6128 and rs6133 SNPs of the *SELP* gene associated with PDR progression, in contrast to rs3917779, which showed a strong association with PDR [110].

#### 3.1.2. *MTHFR*

MTHFR is a rate-limiting enzyme coded by the *MTHFR* gene involved in the remethylation of homocysteine to methionine. The two most studied and reported polymorphisms of the *MTHFR* gene are rs1801133 (a point mutation at C677T resulting in valine to alanine substitution at 222 amino acid chain position) and rs180131 (another point mutation at A1298 resulting in alanine to glutamine substitution at 429 amino acid position). Notably, a point mutation at the C677T position is linked to lower enzymatic activity, hence higher levels of homocysteine, and thereby results in hyperhomocysteinemia. Elevated levels of homocysteine are known to induce endothelial dysfunction, arterial stiffness, atherosclerosis, and retinopathy in both T1D and T2D. A comprehensive meta-analysis including the data reported between 1996 and 2016 by Luo et al. showed the association between *MTHFR* gene variants and DR in two sets of populations (Asian and non-Asian) [95], including a total of 1747 DR cases and 3146 controls, including healthy and diabetic individuals free of DR. Their meta-analysis suggested a strong correlation between rs1801133 or C677T polymorphism and DR in T2D. Interestingly, the Asian population reflected a strong association between the rs1801133 variant and DR regardless of diabetes type. On the other hand, few clinical studies have reported the rs1801131 and rs1801133 polymorphism and DR correlation, but, due to a small sample size, sample heterogeneity, and environmental factors, this association is less comprehensive [94,96,97].

#### 3.1.3. *NVL* and *CRP*

The nuclear VCP-like protein-2 or nuclear valosin-containing protein-like 2/NVL2 is a member of the nucleolar AAA-ATPase family participating in ribosome biogenesis and telomere assembly. The *NVL* gene encodes NVL2, which is widely expressed in the retina. In 2018, a multiethnic genome-wide association study was performed on a large cohort (over 43,000 subjects, including European, Asian, Hispanic, and African American cohorts) [99]. This was the first ever study that showed an *NVL* gene polymorphism and its association with DR. Notably, rs142293996 was the most significant genetic variant for the European patients with extreme DR; however, without genome-wide significance after meta-analysis with replication cohorts.

The *CRP* gene encodes for C-reactive protein (CRP), a plasma marker for inflammation. CRP is produced by the liver, biologically activating the complement system, and is known to be upregulated in systemic inflammation, cardiovascular diseases, and diabetes as well as diabetic retinopathy [124]. The gene variant rs2808629 for *CRP* was shown to be associated with the susceptibility to develop diabetic retinopathy in T2D patients for over 10 years. Notably, this study was performed in Shanghai; therefore, it is limited to a geographically restricted population. A recent study has highlighted rs1205 gene polymorphism for the *CRP* gene, which is associated with increased plasma CRP levels and is correlated with retinopathy and hypertension in T1D patients [64].

#### 3.1.4. *VEGF*

VEGF is coded by the *VEGF* gene present on chromosome 6 in humans. VEGF is considered one of the determinants of DR development and progression. It is produced by various cell types of the retina, and several studies have shown markedly elevated levels of VEGF in aqueous and vitreous fluids of patients with proliferative DR [125,126,127]. Elevated levels of VEGF in the vitreous increase retinal vascular permeability and neovascularization, contributing to DR development [128]. Awata et al. identified six polymorphisms of the VEGF gene: G(−1877)A, T(−1498)C, G(−1190)A, and G(−1154)A in the promoter region and C(−634)G and C(−7)T in the 5′-untranslated region (UTR) [112]. Of these six SNPs, they reported that the C(−634)G or rs2010963 SNP was a risk factor for developing DR in T2D. Different results were found by Uthra et al. in an Indian cohort, where no strong correlation was found between C(−634)G and DR in T2D patients; however, the authors found that this polymorphism increased the risk of developing DR in patients with microalbuminuria [115]. In the Chinese population, promoter region polymorphism was associated with the risk of developing DR in T2D patients [117]. An association between *VEGF* polymorphisms and altered DR risk was also reported for different ethnic groups, but with notable inconsistencies [113,114,129,130,131]. A systematic meta-analysis in 2014 showed a significant relationship between the rs3025039 (+936C/T) polymorphism and DR in Asian as well as overall populations, and a significant association was also found between the rs833061 (−460T/C) polymorphism and susceptibility to developing DR [132]. A recent meta-analysis showed that the rs2010963 SNP is associated with NPDR in the Asian population and the rs2010963 SNP with PDR in the total population (either Asian or Caucasian). Also, the rs833061 polymorphism was associated with PDR in the Asian population, whereas rs699947 polymorphism was associated with NPDR in the total population and with PDR in the Asian population [119]. Interestingly, a recent study uncovers two SNPs for *VEGF* rs25648 and rs3025039 with a reduced risk for DR development, highlighting a protective candidate locus for personalized interventions [133].

#### 3.1.5. *IL-6*

Interleukin-6 is a pleiotropic proinflammatory cytokine encoded by the IL-6 gene present on chromosome 7. Increased levels of IL-6 in aqueous humor or vitreous humor samples have been reported in diabetic retinopathy [134,135,136]. The genetic polymorphism studies are limited; however, in 2015, Lu et al. reported that two SNPs from the promoter region of the *IL*-6 gene (rs1800795 and rs1800796) were associated with the elevated levels of both IL-6 mRNA and protein. [87]. They also suggested that these SNPs might point toward a relatively high risk for T2D patients suffering from PDR in the Chinese population. Contrarily, a recent meta-analysis showed no possible correlation between the rs1800795 and rs1800796 SNPs and the risk of developing DR [88]. Hence, more genetic studies are needed to evaluate the *IL*-6 gene polymorphism and DR incidence.

#### 3.1.6. *eNOS*

Endothelial nitric oxide (NO), synthesized by eNOS, is an endogenous vasodilator that plays a vital role in maintaining the normal physiological state of microvasculature. Several studies have reported increased levels of NO in T2D patients with DR compared to individuals without DR, suggesting its association with the DR incidence [68,137]. Recent research has shown that the *eNOS* polymorphism may affect eNOS activity and thereby could affect the development and progression of DR. The human *eNOS* gene is located on chromosome 7q35-36. Bazzaz et al. showed an association between gene variant rs2070745 and DR in T1D patients [67]. A recent meta-analysis study covering data from 7838 Caucasian subjects showed that the intron 4a allele of *eNOS* 4a/b is a risk factor for developing DR in T2D patients [73]. This gene polymorphism arises from the 27-base pair variable number of tandem repeats (VNTRs) located in intron 4 of the *eNOS* gene. Additionally, there are studies among different ethnic groups suggesting the heterogeneity among the population. In the Greek T2D population, an association between rs1799983 or *eNOS* G894T was associated with DR, while no role for rs2070744 or *eNOS* T786C [72]. Contrarily, in Chinese patients, a strong correlation has been noted between rs2070744 and DR [138]. Surprisingly, Hassan et al. reported that the C allele of the *eNOS* T786C polymorphism could be protective against DR development in T2D patients [74].

#### 3.1.7. *AR* and *PAI-1*

Aldose reductase is a rate-limiting enzyme of polyol pathways, converting D-glucose into sorbitol and galactose into galactycol. Common polymorphism in the regulatory region of the *AR* gene has been reported to be associated with microvascular complications of DM; however, several studies have reported inconsistencies, possibly due to ethnic differences. The most reported variation is the C(-106T) polymorphism. A recent meta-analysis covering 23 studies by Lin et al. [59] found that there is a significant correlation between *AR* gene C(−106T) polymorphism and DR susceptibility in T1D patients geographically restricted to East Asian and Middle Eastern populations.

The *PAI-1* gene is also located on chromosome 7q21.3-q22. It is a serine-protease inhibitor and its higher activity is associated with atherosclerosis and thromboembolism. The promoter region of the *PAI-1* gene contains a common -675 4G5G polymorphism that may affect its basal and inducible expression. A recent case-control and meta-analysis study by Dastgheib et al. [102] showed that 4G5G polymorphism is associated with both diabetic nephropathy and DR in Asians and Caucasians, respectively. Another SNP for the *PAI-1* gene, rs1799768, has shown no significant association with the development and progression of DR in T2D patients.

#### 3.1.8. *ACE*

The *ACE* gene is present on chromosome 17q23.3, and more than 160 polymorphisms have been reported for this gene [139]. ACE converts angiotensin I to angiotensin II, a vasoconstrictor that induces the expression of VEGF, leading to angiogenesis, thereby making *ACE* an interesting gene target for DR development and progression [140]. An insertion/deletion (I/D) polymorphism of a 287-base pair Alu repetitive sequence at intron 16 (rs1799752) has been thoroughly studied for the *ACE* gene among several ethnic groups over time [141]. In Caucasian subjects with T2D, no correlation between the *ACE* I/D polymorphism and susceptibility to develop DR was observed [142]. Lu et al. reported that D/D allele carriers have higher levels of ACE in their serum when compared to I/I or I/D genotype in the Chinese population [46]. Some recent studies have highlighted ethnic differences between the *ACE* polymorphism and DR susceptibility. In a Mexican T2D cohort, the higher frequency of I/I polymorphism was associated with DR development [51]. In middle-aged Indian T2D patients, the authors did not find any correlation between I/D gene polymorphism and DR; however, there was a correlation between *ACE* I/D polymorphism and severity of DR in these patients [53]. Interestingly, *ACE* polymorphism has also established its role in T1D, where Zafar et al. showed that *ACE* I/D polymorphism correlates with DR development, with the prevalence of D and I alleles as 57.5% and 42.5% in DR patients, respectively; followed by 66% and 34% in T1D, and finally 69% and 31% in control subjects. Authors also suggested that the *ACE* gene polymorphism may not have influenced the progression of DR [52]. Contrarily, a most recent meta-analysis combining published data from 1992 to 2022 showed no significant correlation between *ACE* gene I/D polymorphism and DR development and progression in T2D [50].

#### 3.1.9. *ApoE* and *ICAM-1*

Present on chromosome 19, *ApoE* has three major alleles (E2, E3, and E4) and six possible genotypes (E2/2, E2/3, E2/4, E3/3, E3/4, E4/4). The *ApoE* gene codes for apolipoprotein, which plays an important role in carrying lipids in the blood vessels and maintaining blood lipoprotein levels. Some studies suggested an association between *ApoE* variants and T2D [143], in addition to its polymorphic roles in coronary heart diseases, ischemic cerebrovascular disease, and Alzheimer’s disease. However, no significant association between *ApoE* gene polymorphism and DR development and progression has been reported. [54,55,56]. One recent case-control study from the Czech Republic showed that the *ApoE* variant rs429358 is protective against DR development in female T2D subjects. [57]. They stated that there was no association between *APOE2/E2* and *APOE2/E3* and T2D; however, carriers of at least one allele of *ApoE4* (rs428358) were protective against T2D-associated retinopathy.

The *ICAM-1* gene, also present on chromosome 19, codes for intercellular adhesion molecule-1 (ICAM-1). ICAM-1 is a member of the adhesion immunoglobulin superfamily expressed on the surface of leukocytes, endothelial cells, and epithelial cells. Concerning DR, it promotes the adhesion of infiltrating immune cells to the endothelium, leading to perivasculature infiltration. *ICAM-1* 469 (K/K, K/E, E/E) genotype KK was shown to be a genetic risk factor for retinopathy in T2D. However, there have been inconsistencies with the *ICAM-1* gene polymorphism and DR incidence. For example, a recent study from India showed no significant association between *ICAM-1 469* (rs5498) with sight-threatening DR in T2D patients. A meta-analysis from 2018 also showed similar results, with no association between *ICAM-1* gene variants and DR in T2D patients [86]; therefore, more conclusive studies are needed to elucidate the genetic role of the *ICAM-1* gene in DR.

In summary, genetic polymorphism certainly has a strong impact on the disease outcome as well as on the diagnosis of DR, and Table 1 highlights the genes discussed above in addition to several other genes reported to be associated with DR. However, most of these studies are confined to a specific geographical location or races or ethnic groups, resulting in inconsistencies. In contrast, from the viewpoint of precision medicine, these inconsistencies may serve as an asset to highlight population diversity and enhance the accuracy of DR diagnosis. Moreover, large-scale international collaboration to examine SNP data from various regions worldwide could strengthen the current understanding of the DR.

## 4. Lifestyle and Environmental Factors

It is widely recognized that insulin resistance, which results in poor glycemic control, is a key mechanism in the development of T2D. As such, poor glycemic control has been broadly defined in the literature as a clinical risk factor for DR onset and progression [144,145,146]. We will examine two key and adjustable lifestyle factors that greatly affect glycemic control: diet and exercise. Additionally, it has been shown that environmental factors, such as air pollution and volatile organic compound (VOC) exposure, increase the risk of T2D [147,148,149,150]. These factors can then be used, in conjunction with genetic information, racial demographics, and disease phenotype, to prepare specially tailored prevention methods and treatment strategies for precision medicine.

### 4.1. Impact of Lifestyle Factors on DR Risk

It is important to note that exercise, diet, and waist circumference are known to be mediating effectors of HbA1c, a measure of glycemic control. In other words, these lifestyle factors, regardless of whether they are independent of each other, may influence glycemic control, which is known to affect DR diagnosis and progression [151,152,153].

#### 4.1.1. Physical Activity

Low physical activity (PA) is a known modifiable risk factor for T2D, and it has been shown that PA can improve glycemic control in both T1D and T2D [154,155]. A systematic review and meta-analysis by Ren et al. analyzed 22 studies to evaluate the association of physical activity with the risk of DR and noted a modest but significant reduction in relative risk (RR), found to be 0.94 (95% C.I. of 0.90–0.98), representing a relative risk reduction of 6% from the pooled results [156]. Additionally, Ren et al. performed covariate analysis to investigate the impact of PA on VTDR. There were seven studies that included VTDR as a participant variable. RR of PA for VTDR was found to be 0.89 (95% C.I. of 0.8–0.98) [156]. Limitations of this meta-analysis include substantial heterogeneity among the included studies, as indicated by a Cochran’s Q test *p*-value of <0.001 and an I^2^ statistic of 78.9%, suggesting that most of the variability in effect estimates was due to differences between studies rather than chance. Meaning that population characteristics, study criteria, and variables that were accounted for in multivariate analysis (like HbA1c, diabetes duration, age) were not consistent between studies. The heterogeneity of these studies limits our interpretation of the results and further complicates our understanding of the impact of PA on DR independent of glycemic control. While this meta-analysis presents early evidence for a possible link between PA and DR, future research is needed to control for HbA1c.

A more recent study by Li et al. analyzed data from 3482 participants in NHANES to evaluate the influence of lifestyle factors on DR in adults aged 18–64. Multivariate analysis adjusted for gender, age, race/ethnicity, diabetic duration, diabetic neuropathy, cardiovascular disease, HbA1c, blood pressure, and lipids. The adjusted odds ratio was reported as 0.64 (95% C.I. 0.35–0.92), showing that greater physical activity was associated with a decreased prevalence of DR [157]. Another study analyzed a combination of lifestyle factors and their influence on DR risk, and found that individuals with four or more low-risk lifestyle factors (non-smoking, healthy bodyweight, moderate to vigorous PA, high-quality diet, and moderate alcohol consumption) had a substantially lower RR of diabetic retinopathy compared to T2D individuals with zero low-risk lifestyle factors (multivariate adjusted RR = 0.76, 95% C.I. = 0.57–1.01) [158]. It is important to note, though, that this result was deemed “borderline” significant, as the 95% C.I. for RR crosses 1. Overall, PA may be an important factor to consider when determining individual risk and prognosis for DR.

#### 4.1.2. Diet

Diet is a well-known factor that influences the risk of T2D. While high-calorie diets lead to weight gain and, subsequently, insulin resistance, individual dietary components also play a role in the onset of T2D, independent of body mass index (BMI) [159,160]. Here, we will explore the influence of diet on DR risk.

Several studies report that the Mediterranean diet may have a protective effect against the development and progression of DR [161,162,163]. The Mediterranean diet (MedDiet) consists mainly of vegetables, fruits, nuts, olive oil, and cereals with a moderate amount of poultry and fish and a small amount of processed red meats [164]. The Mediterranean diet is rich in fatty acids, characterized by a high ratio of monounsaturated fatty acids (MUFA) to saturated fatty acids (SFA). It is also abundant in vitamins and other antioxidants, contributing to its health-promoting properties [165]. Separately, intake of fruits and vegetables as well as vitamin C and carotene has been found to display substantial reductions in the risk of DR onset, even when controlling for HbA1c [166]. Bryl et al. summarize the literature surrounding the MedDiet and its impact on DR and describe how omega-3 fatty acids from fish and antioxidants from fruits and vegetables found in the MedDiet may confer protection against DR onset [161]. Conversely, poor nutrition is associated with an increased risk of DR. It was found that saturated fatty acids (SFAs), found mainly in red meat and dairy products, increase the risk of DR in T1D individuals [167]. A recent study by Liu et al. examined the diets of individuals with DR and without and then classified their diets according to the dietary inflammatory index (DII). This model weighs the inflammatory character of individual dietary components (such as fat and vitamin content) and produces a DII score. Liu et al. found that individuals with higher DII have a significantly higher risk for DR, even when controlling for HbA1c, BMI, and other systemic factors (OR 1.38, C.I. = 1.06–1.81) [168].

Diet is an essential component of DR risk, and it may be an important factor to consider for a better understanding of individual disease risk. Generally, a diet resembling the Mediterranean diet confers protective benefits that seem to extend beyond improved glucose control. Conversely, a diet that is high in SFAs, ultraprocessed carbohydrates, and trans fats is proinflammatory, ultimately increasing disease risk. This information can guide earlier detection efforts in at-risk patients and support intensified counseling on the benefits of an anti-inflammatory diet for preventing DR.

### 4.2. Impact of Environment on DR Risk

Recent studies have demonstrated that air pollution (defined as fine particulate matter less than 2.5 microns in diameter (PM_2.5_) or coarse matter of diameter 2.5 microns–10 microns (PM_2.5-10_)) may play a significant role in the development of DR. A study published in China by Shan et al. showed that for each 10 µg/m^3^ increase in PM_2.5_, the adjusted OR was 1.41 (95% C.I. 1.27–1.57) for DR [169]. While the authors adjusted the multivariate model for a range of systemic factors known to influence DR risk, they were unable to control for HbA1c, so it is unclear whether the pollution effect is mediated by poor glycemic control. Likewise, researchers in Taiwan found that for every 10 µg/m^3^ increase in PM_2.5_, the adjusted OR was 1.29 (95% C.I. 1.11–1.50), and every 10 µg/m^3^ increase in particulate matter of 2.5 micron–10 micron diameter resulted in an adjusted OR of 1.37 (95% C.I. 1.17–1.61) [170]. Other studies have shown similar results relating to fine and coarse PM [171,172]. Additionally, analysis of NHANES data found that VOCs, which are commonly found in detergents and cleaning agents, increase the risk of DR [173].

Prior studies have shown that fine PM and coarse PM increase the risk of T2D, independent of DR. While the studies by Shan et al. and Pan et al. show that fine and coarse PM increase the risk of DR in an East Asian cohort, neither study controlled for HbA1c in their multivariate regression model. With additional research, environmental factors may be used to stratify individual disease risk.

## 5. Biomarkers for DR

DR’s natural history is asymptomatic and slowly evolving [174]. Biomarkers for DR, found in the serum, aqueous humor, vitreous, and imaging, could provide early information for DR risk and progression, and could potentially be used to optimize treatment selection for individual patients. Anti-VEGF injections, while a promising therapeutic for the treatment of PDR and DME, fail in a subset of patients who receive treatment [175]. Additionally, as we have described in a previous section, DR has variable phenotypic presentations, which can be further elucidated with advanced biomarkers for DR diagnosis, progression, and treatment selection. We will separate these biomarkers by the following location and technique in which they are found: aqueous humor, vitreous, and serum.

### 5.1. Vitreous Humor

Several studies have reported that interleukin-6 (IL-6), a proinflammatory cytokine, and ICAM-1 are found to be elevated in the vitreous of individuals with DR and DME [135,176]. Additionally, VEGF has played a major role as a target for PDR and DME therapy since it was first shown to be elevated in the vitreous of individuals with PDR [125]. Furthermore, elevated vitreous VEGF levels correlate with worsened disease severity in both DR and DME [177,178,179,180,181]. While vitreous VEGF, IL-6, and IL-8 certainly correspond to disease severity [182], and can therefore be used as a biomarker in this manner, we were not able to find any studies predicting treatment response to anti-VEGF therapy specifically in individuals with DR, PDR, or DME. However, a few studies report correlation analysis for vitreous biomarkers in predicting outcomes of pars plana vitrectomy (PPV), a surgical procedure to treat complications of PDR [183]. This is likely due to the invasive nature of vitreous sample collection; therefore, samples are only taken from patients who are already undergoing surgery. A study from 2014 found that vitreous VEGF levels positively predicted future progression of PDR after surgery (OR 1.539, *p*-value = 0.036) [184]. It is important to note that in this study, PDR progression post-surgery was defined anatomically, namely by the presence of vitreous hemorrhage, fibrovascular proliferation, and tractional retinal detachment.

In a more recent paper published in Jordan, Al-Dwairi et al. analyzed the prognostic value of vitreous levels of VEGF and platelet-derived growth factor (PDGF) in PDR participants undergoing PPV. First, the authors compared VEGF and PDGF levels at baseline between a PDR group and a non-DR group undergoing vitrectomy and found significantly elevated VEGF and PDGF levels in the PDR group, as expected [185]. Additionally, this group analyzed best-corrected visual acuity (BCVA) prior to and after vitrectomy and found that higher baseline vitreous VEGF levels did not correlate with BCVA three months from surgery, but increased levels of baseline PDGF seemed to offer a strong predictive value in positive treatment outcome for individuals with PDR [185]. It is important to note that the authors in this study analyzed a different outcome than Wang et al. BCVA, which shows functional outcome in patients, measures the objective improvement in individuals’ vision, while anatomical outcomes do not always correlate with perceived improvement for the patient.

Research on vitreous biomarkers is somewhat limited due to the invasive nature of sample procurement. While inflammatory and angiogenic biomarkers such as IL-6, IL-8, VEGF, and PDGF have been shown to correlate with disease severity, there are limited studies that correlate these biomarkers with treatment response. Due to the nature of sample collection, there are a few studies that show vitreous VEGF levels as a predictor for treatment success of PPV for PDR, with varying results based on the outcome analyzed. While Al-Dwairi et al. found that baseline PDGF predicts PPV outcomes, further research is needed to assess the predictive ability of vitreous VEGF and to confirm vitreous PDGF.

### 5.2. Aqueous Humor

Several studies report a significant, positive correlation between levels of IL-6 and VEGF in aqueous humor and vitreous [186,187]. This is advantageous, as aqueous sample collection is much less invasive and can be obtained in the exam room [188]. A multicenter prospective cohort study by Hillier et al. showed that elevated baseline levels of VEGF in aqueous humor were associated with a worse treatment response with Ranibizumab, an anti-VEGF drug [189]. Aqueous samples were taken at the trial start point and analyzed for a panel of proinflammatory and anti-inflammatory cytokines. After a series of anti-VEGF injections over the course of three months, another sample was procured, and cytokine concentration was determined. Additionally, treatment outcomes were defined by best corrected visual acuity, change in central macular thickness, and change in macular volume. Anti-VEGF treatment was associated with a lower VEGF concentration at the study endpoint, as expected. Additionally, after correcting covariates, the authors found that higher baseline VEGF concentration was associated with an increase in central macular thickness and macular volume as assessed by OCT, while ICAM-1 was negatively correlated with macular volume but not central macular thickness [189]. Neither VEGF nor ICAM-1 baseline levels were associated with BCVA after 3 months.

A retrospective case study by Felfeli et al. analyzed 41 eyes with center-involving DME and classified them as responders (significant decline in central subfield thickness and macular volume) or nonresponders receiving anti-VEGF therapy [190]. While there was no significant difference in baseline aqueous VEGF concentration at the start of care, the responder group had significantly lower concentrations of VEGF compared to nonresponders after two months (two injections) of therapy. Additionally, ICAM-1 concentration significantly decreased in the responder group after two months of treatment [190]. While this study did not show biomarker prediction for treatment response, it does provide another variable to measure treatment response.

In the largest analysis of IL-6 in diabetic eyes, Manda et al. found that elevated aqueous levels of IL-6 correlated with DR severity (no DR, mild NPDR, and PDR) as well as increased central field thickness and macular volume [191]. This prospective, controlled trial provides the greatest evidence that aqueous IL-6 may be an effective biomarker for DR and DME severity.

### 5.3. Serum

Serum sampling is significantly easier and less invasive than either vitreous or aqueous humor sampling. Therefore, there is a large need to investigate serum biomarkers for DR and DME. As discussed in Section 3, HbA1c is one of the most well-studied known risk factors for DR and DME [144,145,146,151,192]. Therefore, in the right model, HbA1c can be used to stratify DR risk and risk of progression.

VEGF, while primarily localized to the posterior segment of the eye, has been detected in serum. A study by Ahuja et al. found that VEGF was detected in serum and the VEGF level incrementally increased between controls, no DR, NPDR, and PDR (*p* < 0.001) [193]. Similarly, a meta-analysis involving 29 studies found that serum levels of VEGF were statistically higher in PDR than they were in NPDR, and were also higher than in the no DR group [194].

Several groups have also detected IL-6 in serum, with concentrations correlating with DR severity [195]. In a prospective case-control study, Kaviarasan et al. similarly found that IL-6 levels reflected disease severity [196]. The ability to detect biomarkers in serum offers a less invasive approach that could improve participation and clinical utility.

### 5.4. Circulating Angiogenic Cells

Circulating angiogenic cells (CACs) are bone-marrow-derived cells known to play a critical role in the pathogenesis of DR [197,198,199]. Our decades of research demonstrate that these cells could help in the repair of retinal microvasculature through paracrine and autocrine mechanisms [200,201,202], and efforts have been made to treat retinal vascular lesions in both preclinical and clinical studies [203,204,205,206]. Due to the striking similarity of this cell type with microvasculature, they could serve as biomarkers for DR, and molecular signatures for transcriptome and miRnomics could help in understanding overall vascular health, which, along with other systemic factors, can further improve precision in DR diagnosis [207,208].

## 6. Conclusions and Future Directions

**Figure 2:**
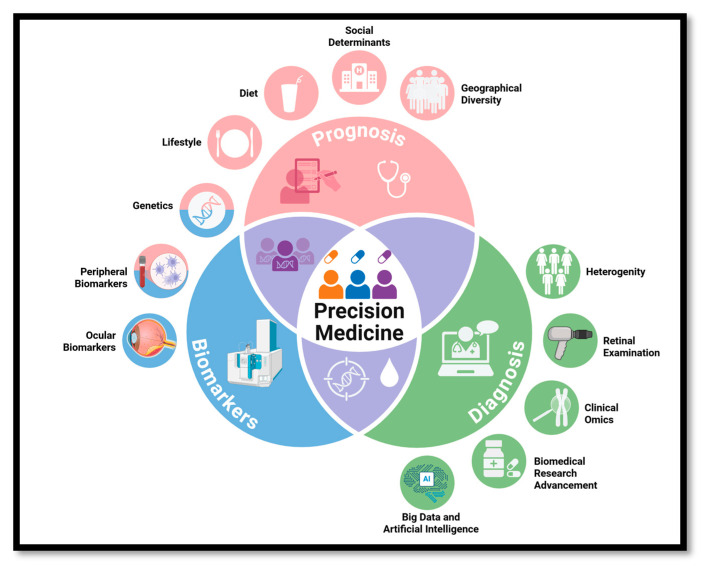
Precision medicine approaches in DR: current status and future perspective.

As discussed earlier, various factors such as diagnosis, biomarkers, and prognosis play a crucial role in developing precision in DR management and treatment (Figure 2). The nature of diabetes (T1D vs. T2D), genetics (e.g., VEGF polymorphism), race, sex (e.g., ASAB-male), and other comorbidities can form the basis of disease pathology. Along with this, lifestyle factors (e.g., exercise), diet (e.g., Mediterranean diet), and environmental influences (e.g., air pollution) may impact personalized treatment for DR. Rapidly advancing diagnostic techniques and improvements in instrumentation are providing cutting-edge tools for today’s physicians to improve diagnostic accuracy, thereby aiding better prognosis of DR. While genetics help identify key biomarkers in blood, CACs, and the eye itself, there is a need to integrate all these aspects to better characterize individuals with DR for diabetes management and early intervention. The rapidly evolving field of AI has the potential to unify these different aspects of precision medicine in DR, which is poised to advance medical practice significantly.

In ophthalmology, AI systems are already being developed for the early detection of DR and DME. In 2018, years before large language models (LLMs) like ChatGPT came into the mainstream, an autonomous AI detection software for DR was developed by Abràmoff et al. and cleared for FDA approval [209]. The authors of this study showed that their AI system, consisting of an ensemble deep learning framework trained on over a million fundus images, was effectively able to differentiate mild DR from more severe DR based on 45° fundus images with good sensitivity and specificity [209]. This software has been further validated in multiple real-world studies [210,211].

Since the development of this AI application in DR screening, we have seen an explosion of varying AI algorithms for DR and DME screening, staging, and prognosis [212]. Several studies have demonstrated the efficacy of AI models for anti-VEGF treatment prediction in DME for both anatomical response [213] and BCVA [214] with area under receiver operating characteristic curves (AUCs) of 0.9 and 0.8, respectively. Both studies are different in their algorithmic approach; however; Lu et al. trained five supervised ML models and incorporated OCT metrics, along with blood biomarkers (HbA1c, hematologic parameters, total cholesterol) and other systemic factors, and then tested them with a prospective treatment group to analyze the independent predictive ability of each model [213]. In comparison, Liu et al. utilized a heterogeneous ensemble (deep learning convolutional neural networks (CNNs) with ML) to integrate OCT image analysis with clinical data [214]. Similarly, Mondal et al. utilized an ensemble deep learning framework, combining a CNN for image analysis with an ML algorithm for DME disease length and serum VEGF receptor 2, and successfully predicted treatment response with an AUC of 0.89 [215]. These approaches are promising for the future of precision medicine, allowing clinicians to use OCT and biomarker data for treatment response predictions, leading to better patient outcomes and decreased healthcare costs.

Interestingly, a study from Dai et al. showed that their deep learning algorithm was able to detect “time to progression” for patients with DR [216]. They compared several different models and found that the model that only utilized a CNN for image analysis performed better than the “metadata” model, which combined an array of systemic factors with automatic image analysis for an integrated prediction model [216]. The authors then tested their model in a real cohort of patients to evaluate the impact on workflow in the clinic. They found that, if all participants followed the recommended screening interval from the model, the mean screening interval could be extended from 12 months to 31.97 months in diabetic patients with no or mild NPDR [216].

AI can integrate complex data sets for precision medicine for DR. Importantly, these systems may also have the potential to identify important biomarkers through -omics screening, answer patient questions through generative AI chatbots, and screen for an array of different eye diseases [217]. While these applications of AI are still being explored and validated in real-world studies, there is a remaining question of the usability and adoptability by the healthcare system. To integrate these systems into real-world clinical or science practice, the overall impact on healthcare (economics, access, and disease prevention) must be considered. As such, Channa et al. analyzed the potential for autonomous AI eye disease screening compared to eye care provider (ECP) screening and found that their modified workflow could prevent 27,000 Americans from suffering from vision loss over a five-year interval [218], thus demonstrating the potential feasibility of improving vision-loss prevention through an altered clinical workflow.

These breakthroughs in medical research have the potential to transform DR and DME screening, diagnosis, and treatment. Deep learning can detect fundoscopic and OCT features that are invisible to human graders, allowing us to detect patterns beyond human capabilities. Ensemble AI systems, combining image analysis through CNNs and clinical variables through ML algorithms, offer an exciting opportunity for precision medicine in DR and DME management. In the future, phenotypic heterogeneity, individual demographics, lifestyle and environmental factors, genome characteristics, and systematic factors may be explored in combination with AI systems for the development of a true individualized approach to disease management.

Precision medicine is built upon three fundamental elements: genetics, lifestyle, and environment, as shown in the prognosis section. Recent advances in investigating novel biomarkers using high-throughput omics, advances in imaging techniques, and machine learning and artificial intelligence algorithms are redefining the landscape of precision medicine.

## Figures and Tables

**Table 1:** List of genes and genetic variants for diabetic retinopathy.

Genes	Chromosome	SNPs	Type of Diabetes	Type of DR	Geographical Location	References
*ACE*	17	Insertion/deletion (I/D) polymorphism in the ACE gene	T1DM and T2DM	DR	-	[41]
		Insertion/deletion (I/D) polymorphism	T1DM	NPDR and PDR	Vienna	[42]
		Insertion/deletion (I/D) polymorphism	T1DM and T2DM	DR	-	[43]
		Insertion/deletion (I/D) polymorphism	T1DM and T2DM	DR	-	[44]
		Insertion/deletion (I/D) polymorphism	T2DM	DR	Iran	[45]
		Insertion/deletion (I/D) polymorphism	-	PDR and BDR	China	[46]
		Insertion/deletion (I/D) polymorphism	T2DM	DR	Pakistan	[47]
		Insertion/deletion (I/D) polymorphism	T2DM	DR	Asia	[48]
		ACE I/D and AGT M/T gene polymorphisms	T2DM	DR	China	[49]
		ACE gene (I/D) polymorphism (rs1799752)	T2DM	DR	-	[50]
		Insertion/deletion (I/D) polymorphism	T2DM	DR	Mexico	[51]
		Insertion/deletion (I/D) polymorphism	T1DM	DR	Pakistan	[52]
		Insertion/deletion (I/D) polymorphism	T2DM	DR	India	[53]
*APOE*	19	e2/e3/e4 polymorphisms	T1DM	DR	Russia	[54]
		e2/e3/e4 polymorphisms	T1DM and T2DM	NPDR and PDR	Brazil	[55]
		e2/e3/e4 polymorphisms	T2DM	DR	USA	[56]
		rs429358 and rs7412	T2DM	DR	Czech Republic	[57]
*AR*	7	rs759853	T2DM	DR	Egypt	[58]
		C(-106)T polymorphism	T1DM and T2DM	DR	-	[59]
*CAPN10*	2	SNP-43 1/1	T2DM	DR	Poland	[60]
		SNP43	T2DM	DR	Poland	[61]
*CEP135*	4	rs4865047	T1DM	PDR	Lithuania	[62]
*CRP*	1	rs2808629, rs3093077, rs1130864 and rs2808634	T2DM	DR	China	[63]
		+1846 C>T polymorphism	T1DM	DR	Poland	[64]
*EHD3*	2	-	T2DM	DR	Japan	[65]
*eNOS*	7	eNOS4b/b, eNOS4b/a and eNOS4a/a	T1DM	NPDR and PDR	Parice, France	[66]
		−786*C/T	T1DM	DR	-	[67]
		eNOS4 allele	T2DM	PDR	India	[68]
		−786T/C, the VNTR intron 4 a/b and the 894G/T (Glu298Asp) polymorphisms	T2DM	DR	Brazil	[69]
		T-786C, G894T and 27VNTR	T2DM	DR	Asian Indian	[70]
		eNOS-4b/a polymorphism	T2DM	DR	-	[71]
		T786C and G894T	T2DM	DR	Greece	[72]
		eNOS 4a/b polymorphism	T2DM	DR	-	[73]
		intron 4ab, exon 7 Glu298Asp variant (G894T), and T-786C	T2DM	DR	Jordan	[74]
*FAM18B*	17	rs11871508 (G>A)	-	PDR	-	[75]
*GORAB or SCYL1BP1*	1	rs6427247	T2DM	DR	China	[76]
*GRB2*	17	rs3805931 and rs9896052	T2DM	STDR	Australia	[77]
		rs9896052	T2DM	PDR	Brazil	[78]
*HS6ST3*	13	rs2038823	T2DM	DR	Taiwan	[79]
*ICAM1*	19	469 (K/K, K/E and E/E allele)	T2DM	DR	Japan	[80]
		469E (EE) and G241A	T2DM	PDR	Slovenia	[81]
		K469E (rs5498)	T2DM	DR	India	[82]
		rs1801714	T2DM	NPDR	-	[83]
		rs5498	T2DM	DR	China	[84]
		rs5498	T2DM	PDR and NPDR	China	[85]
		rs5498	T2DM	DR	-	[86]
*IL-6*	7	rs1800795 GC and rs1800796 GG	T2DM	PDR	China	[87]
		rs1800795 and rs1800796,	-	DR	-	[88]
		rs1800795, rs1800796 and rs1800797 rs2069837 and rs2069840	-	DR	-	[89]
*INSR*	19	rs2115386	T2DM	STDR and PDR	China	[90]
*JPH2*	20	rs761207 and rs6031415	T2DM	DR	Taiwan	[91]
*KCNJ11*	11	rs2285676	T2DM	DR	Italy	[92]
		rs5219	T2DM	DR	China	[93]
*MTHFR*	1	C677T polymorphism	T2DM	DR	Japan	[94]
		677C/T polymorphism	T1DM and T2DM	DR	-	[95]
		A1298C	DM	DR	Middle East Countries	[96]
		rs1801133	T2DM	DR	Pakistan	[97]
*MYO5C*	15	rs3751624	T2DM	NPDR and PDR	-	[98]
*MYSM1*	1	rs2811893 and rs12092121	T2DM	DR	Taiwan	[79]
*NPY2R*	4	rs1902491	T1DM	PDR	Lithuania	[62]
*NVL*	1	rs142293996	T2DM	DR	-	[99]
*PAI-1*	7	4G/5G (deletion/insertion) polymorphism	T2DM	DR	Pakistan	[47]
		4G/5G and −844G/A	T2DM	DR	Tunisia	[100]
		rs2070682	T2DM	DR	Greece	[101]
		4G5G polymorphism	DM	DR	-	[102]
*PALM2*	9	rs140508424	T2DM	DR	Japan	[65]
*PLXDC2*	10	rs1571942 (C/T)	T2DM	DR	Taiwan	[79]
*PPARα*	3	rs4253778, rs135539 and rs1800206	T2DM	DR	China	[103]
*PPARγ*	3	Pro12Ala, C1431T, C-2821T, A-2819G	T2DM	PDR	Italy	[104]
		rs1805192, rs709158, rs3856806, rs4684847	T2DM	DR	China	[105]
		rs1801282 (Pro12Ala)	T2DM	DR	Poland	[61]
		rs1801282 C/G and rs3856806 C/T polymorphism	DM	DR	Europe and Asia	[106]
*PTPN1*	20	rs3787345 and rs754118	T2DM	DR	Poland	[61]
*RNLS*	10	rs2296545	T2DM	DR	Poland	[107]
*SELP*	1	rs6128, rs6133, and rs3917779	T2DM	DR	-	[108]
		rs6128	T2DM	DR; PDR	USA	[109]
		rs6128, rs6133, and rs3917779	T2DM	PDR	Iran	[110]
*STT3B*	3	rs12630354	T2DM	DR	Japan	[65]
*TIMP3*	22	−899T/A, −915A/G and −1296T/C	T2DM	PDR	-	[111]
*VEGF*	6	C(−634)G polymorphism	T2DM	DR; PDR	Japan	[112]
		C(−7)T and C(−634)G in the 5′ UTR	T2DM	DR	India	[68]
		I/D polymorphism	T2DM	DR	Lublin	[113]
		−634 (the G/C polymorphism) and −460 (the C/T polymorphism)	T2DM	NPDR and PDR	Poland	[114]
		rs201963 or −634C/G and 936C/T polymorphisms	T2DM	DR	India	[115]
		−160C, −152A (rs13207351), and −116A (rs1570360); +4618 (rs735286) and +5092 (rs2146323)	DM	PDR	Northern European Region	[116]
		rs699947, rs833061, rs13207351, rs2010963, rs833069, rs2146323, rs3025021, and rs3025039	T2DM	DR	China	[117]
		−460T/C and −2578C/A polymorphisms	-	DR	-	[118]
		rs2010963, rs833061 and rs699947	T1DM and T2DM	NPDR and PDR	-	[119]
		rs699947	T2DM	DR	Indonesia	[120]
		−2578C/A (rs699947) and −460T/C (rs833061)	DM	NPDR and PDR	Egypt	[121]
		rs699947 and rs35569394	T2DM	DR	India	[122]
*ZNRF1*	16	rs17684886	T2DM	NPDR and PDR	China	[76]

Abbreviations: SNPs—single nucleotide polymorphisms, T1DM—type 1 diabetes mellitus, T2DM—type 2 diabetes mellitus, DM—diabetes mellitus, DR—diabetic retinopathy, NPDR—nonproliferative diabetic retinopathy, PDR—proliferative diabetic retinopathy, STDR—sight-threatening diabetic retinopathy, ACE—angiotensin converting enzyme, APOE—apolipoprotein E, AR—aldose reductase, CAPN10—calpain 10, CEP135—centrosomal protein, CRP—c-reactive protein, EHD3—Eps15 homology-domain-containing protein 3, eNOS—endothelial nitric oxide synthase, FAM18B—family sequence similarity 18, member B, GORAB—Golgin, RAB6-interacting, SCYL1BP1—SCY1-like 1-binding protein 1, GRB2—growth-factor-receptor-bound protein 2, HS6ST3—heparan sulfate 6-O-sulphotransferase 3, ICAM—intercellular adhesion molecule-1, IL-6—interleukin 6, INSR—insulin receptor, JPH2—junctophilin 2, KCNJ11—potassium inwardly rectifying channel subfamily J member 11, MTHFR-methylenetetrahydrofolate reductase, MYO5C—myosin VC, MYSM1—myb-like, SWIRM, and MPN domains 1, NPY2R—neuropeptide Y receptor type 2, NVL—nuclear VCP-like protein 2, PAI-1—plasminogen activator inhibitor-1, PALM2—paralemmin 2, PLXDC2—plexin-domain-containing 2, PPAR—peroxisome proliferator-activated receptor, PTPN1—protein tyrosine phosphatase nonreceptor type 1, RNLS—renalase, SELP—selectin P, STT3B—STT3 oligosaccharyltransferase complex catalytic subunit B, TIMP3—tissue inhibitor of metalloproteinase-3, VEGF—vascular endothelial growth factor.

## Data Availability

No new data were created or analyzed in this study.

## Abbreviations

DR	Diabetic retinopathy
NPDR	Nonproliferative diabetic retinopathy
PDR	Proliferative diabetic retinopathy
T1D	Type 1 diabetes
T2D	Type 2 diabetes
VTDR	Vision-threatening diabetic retinopathy
DME	Diabetic macular edema
ETDRS	Early treatment of diabetic retinopathy study (ETDRS)
ICDR	International clinical diabetic retinopathy
OCT	Optical coherence tomography
OCT-A	OCT-angiography
NHANES	National Health and Nutrition Exam Surveys
